# Genetic Characterization of Yellow Fever Virus Strain JSS, the Original South American Strain

**DOI:** 10.3390/v18050564

**Published:** 2026-05-15

**Authors:** Madison E. Lee, Clairissa A. Hansen, Jill K. Thompson, Haiping Hao, Nigel Bourne, Alan D. T. Barrett

**Affiliations:** 1 Department of Pathology, University of Texas Medical Branch (UTMB), Galveston, TX 77555, USA; 2 Department of Biochemistry and Molecular Biology, University of Texas Medical Branch, Galveston, TX 77555, USAhahao@utmb.edu (H.H.); 3 Sealy Institute for Vaccine Sciences, University of Texas Medical Branch, Galveston, TX 77555, USA; 4 Department of Pediatrics, University of Texas Medical Branch, Galveston, TX 77555, USA

**Keywords:** yellow fever virus, South America, JSS, Brazilian yellow fever virus

## Abstract

Yellow fever virus (YFV) is divided into seven genotypes, including West Africa II (WAII) and South America I (SAI). The first wild-type YFVs isolated, Asibi (Ghana/1927) and French Viscerotropic virus ([FVV] Senegal/1927), are members of WAII. The first YFV strain isolated in South America, JSS (Brazil/1935), was associated with the last outbreak of urban YF in Brazil and has been insufficiently studied. We utilized Next Generation Sequencing to compare JSS with Asibi, FVV, and other South American YFV strains. SAI strains, including JSS, had higher genetic diversity than WAII strains. Phylogenetic and phylogeographic studies of YFV in South America have revealed the circulation of five lineages within Brazil, termed 1A-1E. JSS was found to be distinct from the five genetic lineages currently recognized in Brazil, and so we termed JSS as the currently sole member of Brazilian linage 1F. a comparison of JSS with all other Brazilian genomes of YFV suggests that lineage 1F appears to have become extinct.

## 1. Introduction

Yellow fever virus (YFV), the causative agent of yellow fever (YF) disease, is the prototypical member of the *Orthoflavivirus* genus. It exists in a transmission cycle involving mosquitoes and non-human primates (NHPs) and remains endemic across 47 countries in tropical regions of sub-Saharan Africa and South America. YF disease can range in severity from a mild febrile illness to a life-threatening disease with hemorrhage and substantial liver damage [1]. YFV is believed to have emerged over 4000 years ago in East Africa, subsequently spreading westwards across the African continent, and then being transported to the Western Hemisphere via the slave trade [2]. It was one of the first characterized viruses, the first arthropod-borne virus (arbovirus) identified, and has been extensively studied as a result of its historical significance [3].

The genome of YFV is 11 kb single-stranded, positive-sense RNA molecule that is translated as a single polyprotein and co- and post-translationally processed to produce a total of 10 proteins: three structural (capsid [C], pre-membrane/membrane [prM/M], and envelope [E]) and seven non-structural (NS) proteins (NS1, NS2A, NS2B, NS3, NS4A, NS4B, and NS5). The structural proteins form the virion, while the NS proteins make up the viral replication complex (RC). Like other RNA viruses, the RC of YFV exhibits low fidelity, with a high mutation frequency of 10^−3^–10^−6^ errors per nucleotide (NT) contributing to the genetic diversity of viral populations [4]. Phylogenetically, YFV can be characterized into seven genotypes (East Africa [EA], East and Central Africa [ECA], West Africa I [WAI], West Africa II [WAII], South America I [SAI], South America II [SAII], and Angola [ANG]) [5,6,7,8]. Phylogenetic and phylogeographic studies of YFV in South America have revealed the circulation of five lineages within Brazil, termed 1A-1E [9].

The first wild-type (WT) YFV strains were isolated from human clinical cases, Asibi (Ghana/1927) and French Viscerotropic virus ([FVV] Senegal/1927), and are members of the WAII genotype. The first South America WT strain JSS was isolated from a human clinical case in Brazil during the 1935 epidemic, which is considered the last reported outbreak of urban YF in Brazil [10,11]. Experiments comparing the virulence phenotypes of the JSS, FVV and Asibi strains in mice showed JSS to be less virulent in terms of a longer average survival time compared to the two WAII strains [11]. However, one limitation of this study was the lack of information regarding the viscerotropism of each strain due to WT YFV only exhibiting neurotropic disease in immunocompetent mice [12]. The three WT YFV strains were also included in studies analyzing the antigenic differences between *orthoflaviviruses* (termed group B arboviruses at that time) [13]. These early experiments led researchers to conclude that the South America strains, including JSS, were antigenically distinct from West African strains.

In the studies described here, we undertook Next Generation Sequencing (NGS) on JSS and seven other strains of WT YFV isolated from different geographic areas within South America to evaluate genetic variation in JSS and other members of the SAI genotype. We also compared the genetic variation in the SAI strains with the prototype WAII genotype WT strains Asibi and FVV. Additionally, we examined AA differences between all strains to identify any lineage-specific amino acids (LSaas). Previous studies using small regions of the YFV genome identified JSS as a phylogenetically distinct member of the SAI genotype [6,7]. The whole-genome studies described here confirm and expand these studies to show that JSS forms its own unique lineage within Brazil YFVs. In addition, JSS had more LSaas (14) than any of the other SAI strains examined, which ranged from 2 to 10, and JSS was more genetically diverse than both WT Asibi and FVV strains.

## 2. Materials and Methods

### 2.1. Viruses

All virus isolates subjected to NGS were described and used in previous publications by our group and can be found in Table 1 [5,6,7,8,14,15]. All sequences used in this study can be accessed through the GenBank accession numbers in Table 1. NGS data for the strains sequenced in this manuscript can be accessed through: PRJNA1312863.

### 2.2. RNA Extraction and Sequencing

RNA was extracted from all three strains using the Qiagen Viral RNA Mini Kit (Qiagen, Hilden, Germany) and sent to the University of Texas Medical Branch (UTMB) NGS Core for genome-wide sequencing. Random hexamer primers were used to construct a library using the TruSeq2 RNA v2 kit via RT-PCR (Illumina, San Diego, CA, USA). Any PCR duplicates were marked and removed prior to random down-sampling to 723 reads/nucleotide via Picard-Tools 3.4.0. deepSNV 1.42.0 and Microsoft Excel were then used to analyze the down-sampled data in terms of Shannon Entropy (SE) and Single Nucleotide Variants (SNVs), respectively, to examine the levels of genetic diversity. The WT Asibi and FVV strains, which have been extensively studied, were included as controls to ensure the validity of sequencing data.

### 2.3. Genotype Consensus Sequence Generation

Consensus sequences for each strain aligned in Geneious Prime (version 2025.1.2) and exported into Microsoft Excel where the data were sorted for differences that were identified and examined for amino acid variation between the consensus sequences of each strain included in our analysis.

### 2.4. Phylogenetic Analysis

Using the consensus sequences generated, a phylogenetic tree was constructed using Clustal Omega (version 1.2.4) and Geneious (version 2025.1.2), using a sample of West Nile virus to serve as the outgroup. This allowed for the visualization of the “age” of all the viruses, and the examination of how close strains/genotypes are related in terms of each other.

### 2.5. Visualization of E Amino Acid Residues

The 3D models of YFV the pre-fusion E protein dimer (6EPK4) and EDIII (2JQM) were obtained from the National Center for Biotechnology Information’s Structure Summary MMDB database (RCSB Protein Data Bank). PyMOL (version 3.1.6.1) was used to mutate the selected South American YFV or JSS LSaas to allow for the visualization of changes.

## 3. Results

### 3.1. Virus Strains Analyzed and Comparison of Consensus Sequences

**Figure 1:**
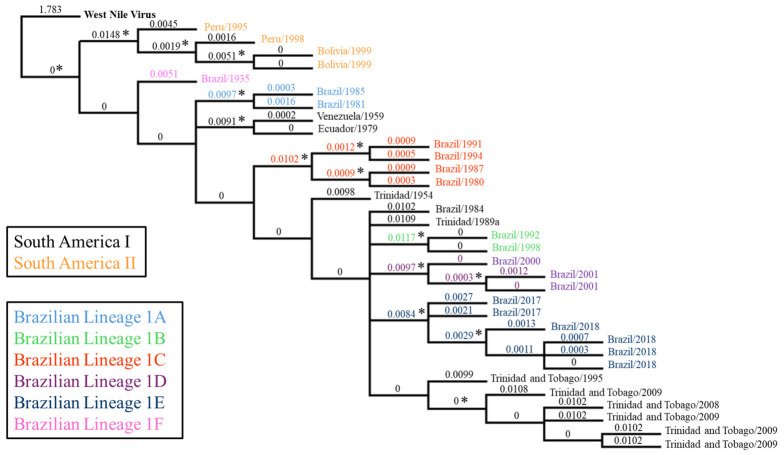
Phylogenetic tree depicting relationships between South American YFVs. The phylogenetic tree was generated using Clustal and Geneious and depicts the relationships between the strains within SAI and SAII, with West Nile virus (WNV) serving as an outgroup. The Brazilian YFV lineages are denoted as light blue (1A), light green (1B), red (1C), purple (1D), blue (1E), and pink (1F). Substitutions per site are listed, with those >80 denoted by asterisks (*).

We examined the sequences of 33 isolates of YFV from South America, (27 strains from GenBank and six (Brazil/1935 [JSS], Ecuador/1979, Venezuela/1959, Trinidad/1954, Trinidad/1989a, and Brazil/1998) sequenced in this study (Table 1)). Because JSS was the central focus of our analysis and was isolated in 1935, we selected the earliest Brazilian isolates we could find in Genbank as well as examples of the five lineages currently circulating in Brazil (1A–1E) [16]. We also included isolates from other countries across South America (Table 1) and two preparations of Ecuador/1979 to confirm that passage history did not bias our results. Consensus sequences were generated for each of the strains sequenced in these studies, aligned and trimmed to contain only the polyprotein region as varying numbers of repeats in the 3′ non-coding region (NCR) could have impacted our dataset [6,14]. A phylogenetic tree was constructed from the strains utilizing West Nile virus (GenBank: HQ596519.1) as an outgroup (Figure 1).

As expected, the topography of the phylogenetic tree showed that the SAI viruses formed a distinct clade from the SAII viruses. In addition, JSS formed a novel lineage distinct from the other SAI viruses and from the five lineages (1A to 1E) recognized in Brazil [5,6,7,8,14,15] (Figure 1). Consequently, we propose that JSS should be considered the sole member of a new Brazilian lineage, which we term 1F. This proposal is supported by the statistical reliability of our dataset (bootstrap values: values > 80 are denoted by asterisks) and substitutions per site. Phylogenetically, JSS was ancestral to all other SAI genotype strains examined, including the members of lineage 1A, the oldest lineage identified to date (Figure 1). None of the three strains (two in lineage 1A plus JSS in lineage 1F) showed evidence of further divergence, suggesting that these old lineages have now become extinct. Nonetheless, examination of the nucleotide (NT) identity of the SAI strains showed that all strains, including JSS, have at least 97% NT identity.

### 3.2. The Genetic Diversity of JSS Is Comparable to Other SAI Genotype Members Although More than the Original WAII Genotype Isolates

**Figure 2:**
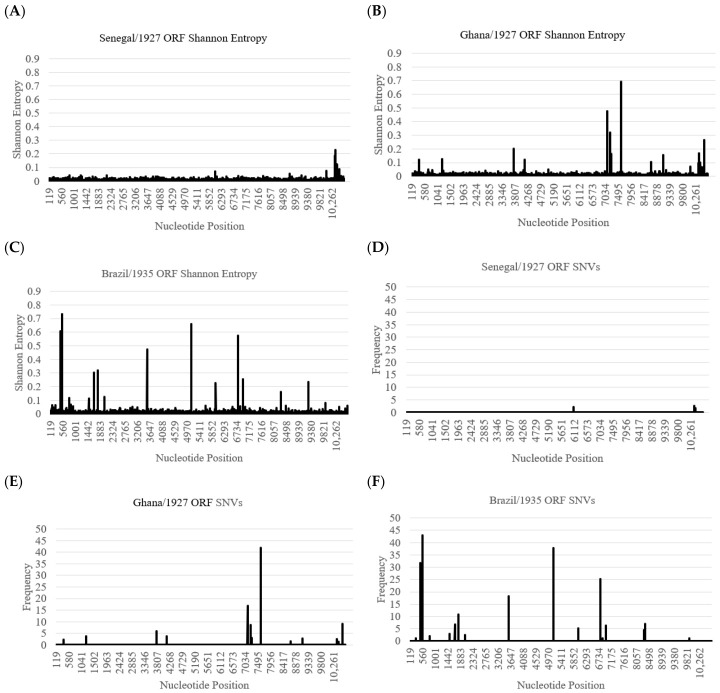
Shannon Entropy and SNVs for Senegal/1927, Ghana/1927, and Brazil/1935. Graphical representation of the Shannon Entropy (SE): (**A**) WAII genotype by Senegal/1927; (**B**) Ghana/1927; (**C**) Brazil/1935 and single-nucleotide variants (SNVs); (**D**) Senegal/1927; (**E**) Ghana/1927; (**F**) Brazil/1935).

The genetic diversity of JSS (Shannon Entropy [SE] 0.0033) was comparable to the six other SAI strains tested (SE ranging from 0.0023 to 0.0039) (Table 2). An examination of Single-Nucleotide Variants (SNVs) revealed a similar pattern ranging from zero to six SNVs > 10% of the viral RNA population, with JSS containing six (Table 2). Separately, JSS was also more genetically diverse than either of the two early WAII genotype WT YFV strains, Asibi (SE: 0.0027 and two coding SNVs > 10.00%) or FVV (SE: 0.0024 and zero coding SNVs > 10.00%) (Table 2 and Figure 2). It is worth noting that we were unable to include strains representing each of the five YFV lineages from Brazil in this analysis. However, we showed that BeAr512943 (Brazil/1998; lineage 1B) has an SE of 0.0029 and zero SNVs > 10.00% [17].

### 3.3. JSS Contains Lineage-Specific Amino Acids Distinguishing It from Other Brazilian Lineages

Examination of the YFV polyprotein sequences of the SAI strains used in this study showed that each of the Brazilian YFV lineages contained LSaas—1A [*n*= 6], 1B [*n* = 10], 1C [*n* = 6], 1D [*n* = 2], 1E [*n* = 2], and 1F [JSS, *n* = 14]—with a combined total of 45 NTs encoding 40 LSaas (Figure 1). Of the 40 codons involved, most (18) of the NT changes occurred in the second position of the codon, with 16 in the first position and (11) in the third NT of the codon (Table 3). In addition, five of the 40 codons contained two NT changes.

**Figure 3:**
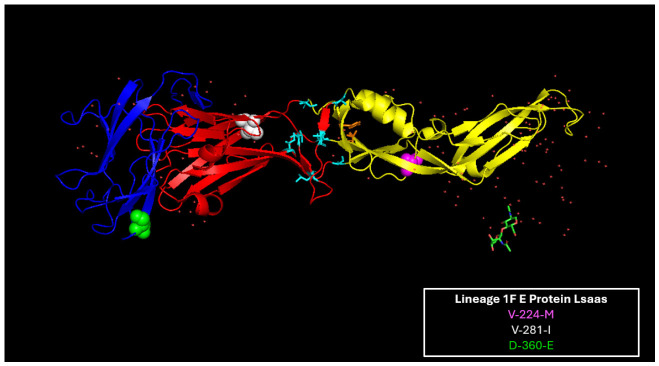
YFV E-protein 1F LSaas. Structural modeling of the first monomer of the YFV E-protein dimer with the JSS LSaas. The domains are denoted in red (EDI), yellow (EDII), and blue (EDIII). E: V-224-M is denoted by magenta; E: V-281-I is represented in white; and E: D-360-E is depicted in green.

LSaas were found in all YFV proteins (Figure 1) but were not equally distributed: 5 were in C, 1 in prM, 3 in E, 4 in NS1, 3 in NS2A, 2 in NS2B, 5 in NS3, 2 in NS4A, 1 in NS4B and 14 in NS5 (Table 3). The unequal ratio of LSaas to number of AAs per protein, plus the greater number of changes occurring in the first and second NTs in the codon suggested that positive selection pressures, as opposed to random mutations, were responsible for each substitution. Significantly, JSS contained 14/40 (35%) of the LSaas identified and was the only lineage that had substitutions in the prM, E, and NS4A (n = two) proteins, suggesting that JSS was subjected to different selection pressures compared to the other Brazilian lineages and that JSS likely failed to adapt to these selective pressures (Figure 3).

### 3.4. JSS and Other WT South American Strains Exhibit Significant AA Differences from African Strains

We compared the polyprotein sequences of Asibi and FVV with JSS and identified 110 AA differences, with changes occurring in every viral protein: (C (7), prM/M (3), E (19), NS1 (11), NS2A (5), NS2B (4), NS3 (9), NS4A (6), NS4B (3), and NS5 (43) (Table 4).

**Figure 4:**
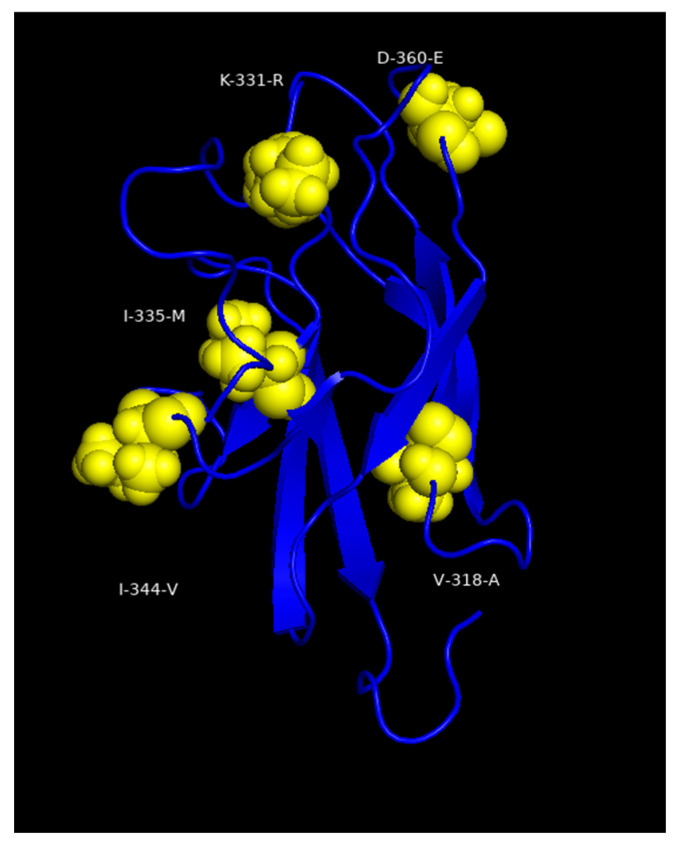
YFV South American (JSS)-specific residues in EDIII. Structural modeling of EDIII with the South American (JSS)-altered amino acids depicted by yellow spheres. The spheres are labeled with white text depicting the canonical amino acid, then the amino acid number, then the altered amino acid.

Further, a more recent Brazilian strain (BeAr512943; Brazil/1998; lineage 1B) differed from Asibi and FVV by 113 AAs but from JSS by only 29 AAs (Supplementary Table S1). Although most differences between JSS, Asibi and FVV involved single AAs, there were eight doublet differences (one in NS1, one in NS3, and six in NS5) and two triplet AA differences (one each in E and NS5). In the E protein, the triplet (E-D270N, E-D271S, E-N272K) occurred in motif 4 of the EDI-EDII hinge region (Table 5) and an alignment of the AAs in motif 4 showed that YFV has a three-residue insertion at E-270-271-272 not found in other mosquito-borne *orthoflaviviruses*. In African genotype strains, the AAs 270-272 triplet is DNN, while for SAI genotype strains it is NSK and for SAII genotype strains it is GSN. In addition, residue 268 is an E for ECA, EA and ANG genotypes while it is a T for the remaining four genotypes (WAI, WAII, SAI and SAIII). The South American (JSS) E protein differs from that of the WAII viruses (Asibi and FVV) by 19 residues, including five residues (26.32%) in EDIII (Figure 4). The NS5 protein differs from the WAII viruses in having the greatest number of AAs (43), which span both the methyltransferase and RNA-dependent RNA-polymerase (RdRp) domains, but none were found at critical residues in the functions of the two domains [18,19,20] (Table 4).

## 4. Discussion

The JSS strain (Brazil/1935) is of historical significance as the first South American strain of YFV. Previous studies using sequences in the prM/E and NS5/3′NCR showed JSS to be phylogenetically distinct from other SAI genotype strains [6,7,21]. Our studies using the entire coding region of the genome extend these observations and confirm that JSS is genetically distinct from other SAI strains in terms of both nucleotide identity and genetic diversity (Table 2). The current literature recognizes five lineages of YFV present within Brazil, which have been termed 1A to 1E [16]. Our phylogenetic studies indicate that JSS forms a divergent and unique Brazilian lineage, which we term 1F (Figure 1).

To investigate the genetic differences that distinguish JSS from other SAI genotype strains, we compared the consensus sequences of each to identify LSaas (Table 3). JSS contained the most NT changes (16; 18.6%) leading to the most LSaas (14) (Table 3) while lineage 1E contained the fewest NT changes (2.33%). These data are consistent with the extensive selection pressures on JSS that possibly contributed to the strain becoming extinct. However, the studies have a number of limitations. First, the exact passage history of the JSS strain used in our studies is unknown since its isolation occurred in 1935 (i.e., before cell culture was developed), and second, we lack SE and SNV data representative of each Brazilian lineage [22] as our data only includes NGS data for JSS and one strain from Brazilian lineage 1B.

One possible explanation for the AA changes in the SAI strains is that they are a response to the selection pressure of adapting to the new NHP hosts and mosquito vectors found in South America [23]. Of the 110 AA alterations between the African YFVs and JSS, 29 occurred in the structural proteins, with 81 in the NS proteins (Table 4). However, 65.5% of the structural protein differences are in the E protein and 53% of NS proteins differences are in NS5. Further, E and NS5 each have a motif of a triplet of differing residues. The triplet in the E protein occurs in a region in which YFV has a 3 AA insertion that is not seen in other *orthoflaviviruses* in motif 4 of the kl β-hairpin within the EDI-EDII hinge region. This region is important for the conversion of the E protein from a dimer to trimer, a process required for the internalization of the virus particle via receptor-mediated endocytosis (Table 5) Interestingly, JSS and all other South American YFVs differ from the African YFVs in this region, with members of SAI differing by three residues (D-270-N, D-271-S, E-N-272-K) and SAII strains differing by two (D-270-G and N-271-S) (Table 5) indicative of a strong selective pressure at this site in YFV in South America. Although there are no reports characterizing the mutations in motif 4 of the EDI-EDII hinge region in YFV, there are studies with other *orthoflaviviruses* that show that determinants of mouse and NHP neuroinvasiveness and other phenotypic properties are found in this motif [24,25,26,27,28,29]. This strongly suggests this motif is worthy of further study in YFV.

Prior studies have shown that Asibi and FVV strains differ from JSS antigenically [13], but the altered antigenic site(s) have not been characterized. The EDIII region of the South American strains contains five AA differences compared to Asibi and FVV (Table 5 and Figure 4). EDIII is known to contain epitopes involved in distinguishing 17D vaccine substrains and to play a crucial role in receptor binding [30,31]. Consequently, we modeled the five AA differences seen in JSS onto the 3D structure of Asibi EDIII (Figure 4) and showed that three of the five changes (V-318-A, K-331-R, and I-335-M) occur in the three β-sheets, suggesting this may contribute to the antigenic differences between the African and South American strains.

In conclusion, we have shown that the first strain of WT YFV, isolated in South America, JSS, is a unique strain that represents the only member of Brazilian lineage 1F, and this lineage has likely become extinct.

## Figures and Tables

**Table 1:** Viruses used for this study. A list of isolates used for this study, including GenBank accession number, strain, taxon, known host information, and known passage history for each virus used. SM = suckling mice brain.

Strain	AA Accession Number	Taxon	Host Information	Passage History
JSS	PX471992	Brazil/1935	Unknown	SM 8, C6/36 1, Vero 1
BeAn510268/TVP21800	QXU67531.1	Brazil/1991	Alouatta sp	Unknown
BeAR378600	AFH35033.1	Brazil/1980	Haemagogus sp	Unknown
BeAr437159/TVP21803	QXU67534.1	Brazil/1985	Haemagogus janthinomys	Unknown
BeH394880	AFH35034.1	Brazil/1981	Homo sapiens	Unknown
P128MC SMB/IVIC	PX246674	Venezuela/1959	Unknown	SM 3, C6/36 1, Vero 1
Ecuador 1337	PX246682	Ecuador/1979	Unknown	Vero 1
BeAR512943	PX246675	Brazil/1998	Unknown	C6/36 1, Vero 1
BeAR513008	AFH35039.1	Brazil/1992	Sabethes sp	Unknown
GML902621	PX246676	Trinidad/1954	Unknown	Monkey 1, C6/36 1
Brazil YFV070FIG	QHB50166.1	Brazil/2018	Homo sapiens	Unknown
Brazil YFV065FIG	QHB50164.1	Brazil/2018	Homo sapiens	Unknown
Brazil YFV072FIG	QHB50167.1	Brazil/2018	Homo sapiens	Unknown
Brazil YFV066FIG	QHB50165.1	Brazil/2018	Homo sapiens	Unknown
PR5951-BeAn845401	ASY08198.1	Brazil/2017	Aotus ozzarae infulatus	Unknown
PR5908-BeAn844641	ASY08195.1	Brazil/2017	Alouatta sp	Unknown
890692 CAREC	PX246684	Trinidad/1989a	Unknown	C6/36 2
BeH422973	AFH35036.1	Brazil/1984	Homo sapiens	Unknown
FIOCRUZ 71530/MG/01	AWK57896.1	Brazil/2001	Homo sapiens	Unknown
BeH622493	AFH35042.1	Brazil/2000	Homo sapiens	Unknown
TR8194/TVP21826	QXU67557.1	Trinidad and Tobago/2009	Coquillettidia venezuelensis	Unknown
TR8183/TVP21825	QXU67556.1	Trinidad and Tobago/2009	Culex spissipes	Unknown
TR7856/TVP21824	QXU67555.1	Trinidad and Tobago/2008	Mansonia titillans	Unknown
TR7796/TVP21823	QXU67554.1	Trinidad and Tobago/2009	Coquillettidia venezuelensis	Unknown
CAREC9515207/ TVP21809	QXU67538.1	Trinidad and Tobago/1995	Haemagogus	Unknown
TVP11767	ADK47994.1	Trinidad and Tobago/2009	Alouatta seniculus	Unknown
OBS2240/TVP21821	QXU67551.1	Peru/1995	Homo sapiens	Unknown
YFV IRF528	XER92105.1	Peru/1998	Homo sapiens	Unknown
Bolivia 88/1999	AVY51405.1	Bolivia/1999	Homo sapiens	Vero E6
Bolivia 88/1999	AHK05343.1	Bolivia 1999	Homo sapiens	C6/36
BeH463676	AFH35038.1	Brazil/1987	Homo sapiens	Unknown
BeH526722	AFH35040.1	Brazil/1994	Homo sapiens	Unknown
BeAR646536	AFH35043.1	Brazil/2001	Haemagogus leucocelaenus	Unknown
14FA	PX246686	Angola/1971	Unknown	SM7, C6/36 1, Vero 4
Couma	PX246683	Ethiopin/1961b	Unknown	SM46, C6/36 2, Vero 1
A-709-A2	PX246689	Uganda/1948a	Unknown	C6/36 1, Vero 1
BA55	PX471990	Nigeria/1987b	Unknown	SM3
ZOBS-7549	PX246681	Bolivia/1999b	Unknown	Vero 1
West Nile	ADT91913.1	New York/1999	Crow	Unknown
Powassan Virus	AAA02739.1	Unknown	Unknown	Unknown
Japanese Encephalitis Virus	AAA81554.1	Unknown	Unknown	Unknown
St. Louis Encephalitis Virus	AAP44973.1	USA	Unknown	Unknown
Rocio Virus	ATG32103.1	Brazil/1975	Homo sapiens	Unknown
Dengue—1	AIU47321.1	USA: Hawaii/1944	Homo sapiens	Unknown
Dengue—2	AAC59275.1	Unknown	Unknown	Unknown
Dengue—3	AAA99437.1	Unknown	Unknown	Unknown
Dengue—4	AAX48017.1	Unknown	Homo sapiens	Unknown

**Table 2:** Shannon Entropy and SNVs of each YFV sequenced by the authors. The SE, SNVs > 1.00%, SNVs > 5%, SNVs > 10%, and coding SNVs > 10% of each virus preparation are listed individually for each SAI strain; also included are Ghana/1927 (Asibi) and Senegal/1927 (FVV). The average and standard deviation of each genotype is listed in the corresponding columns.

	Strain Name	Taxon	Shannon Entropy	SNVs > 1%	SNVs > 5%	SNVs > 10%	Coding SNVs > 10%	Average Shannon Entropy per Genotype	Std.Dev Shannon Entropy per Genotype	Average SNVs > 10% per Genotype	Std.Dev SNVs > 10% per Genotype
South American I	1337	Ecuador/1979	0.0032	7	0	0	0	0.0031	0.0005	2.17	2.99
	P128MC	Venezuela/1959	0.0036	13	7	6	6				
	GML902621	Trinidad/1954	0.0033	7	1	1	1				
	BeAR 512943	Brazil/1992a	0.0029	7	1	0	0				
	CAREC 890692	Trinidad/1989a	0.0023	2	1	0	0				
	JSS	Brazil/1935	0.0033	17	10	6	5				
West Africa II	FVV	Senegal/1927	0.0024	5	0	0	0	0.0026	0.0002	1.00	1.41
	Asibi	Ghana/1927	0.0027	16	5	2	2				

**Table 3:** Lineage-specific amino acid changes exhibited by SAI YFVs. The lineage-specific amino acids for each isolate are listed in the corresponding columns. The protein, conical amino acid is listed before the amino acid number, followed by the lineage-specific amino acid. Also included are the altered nucleotide (NT) numbers, the original codon, the altered codon, and if the change appears to be a result of wobble or selection pressure.

Lineage	LSAA	Codon	LSAA Codon		Lineage	LSAA	Codon	LSAA Codon		Lineage	LSAA	Codon	LSAA Codon
**1A**	C: V-4B-I	GTT	ATT		1B	NS1: K-119-R	AAG	AGG		1C	NS1: N-30-S	AAC	AGC
**1A**	NS1: S-284-N	AGT	AAC		1B	NS2A: M-119-I	ATG	ACG		1C	NS2A: V-178-I	GTT	ATT
**1A**	NS3: A-434-S	GCT	TCC		1B	NS2A: V-147-A	GTC	GCC		1C	NS2B: R-54-K	AGG	AGC
**1A**	NS5: P-152-S	CCA	TCA		1B	NS3: R-444-K	AGG	AAA		1C	NS2B: F-123-L	TTC	CTC
**1A**	NS5: I-258-V	ATC	GTC		1B	NS3: T-562-M	ACG	ATG		1C	NS5: R-95-K	AGA	AAA
**1A**	NS5: T-642-A	ACG	GCG		1B	NS4B: I-20-T	ATT	ACT		1C	NS5: V-132-I	GTC	ATC
					1B	NS5: I-401-M	ATA	ATG					
					1B	NS5: I-728-V	ATT	GTT					
					1B	NS5: V-739-I	GTT	ATT					
					1B	NS5: L-869-F	TTG	TTT					
													
**Lineage**	**LSAA**	**Codon**	**LSAA Codon**		**Lineage**	**LSAA**	**Codon**	**LSAA Codon**		**Lineage**	**LSAA**	**Codon**	**LSAA Codon**
**1D**	NS1: P-332-S	CCT	TCT		1E	C: A-79-V	GTT	GCT		1F	C: S-25-N	AGC	AAC
**1D**	NS5: K-804-R	AAA/ AAG	AGA		1E	C: K-82-R	AAG	AGG		1F	C: S-36-N	AGT	AAC
										1F	prM/M: V-25-I	GTT	ATT
										1F	E: V-224-M	GTG	ATG
										1F	E: V-281-I	GTC	ATC
										1F	E: D-360-E	GAC	GAG
										1F	NS3: Y-20-H	TAT	CAT
										1F	NS3: K-106-R	AAA	AGA
										1F	NS4A: M-67-T	ATG	ACA
										1F	NS4A: V-119-I	GTT	ATT
										1F	NS5: R-2-S	AGA	AGT
										1F	NS5: A-276-T	GCA	ACA
										1F	NS5: T-645-S	ACC	AGC
										1F	NS5: K-702-R	AAA	AGA

**Table 4:** Amino acid differences between South American (JSS) and West African YFVs (Asibi and FVV). The 110 differing amino acids exhibited by JSS as compared with Asibi/FVV. The residues are divided by protein (columns), with the conical amino acid being listed before the amino acid number, followed by the South American amino acid.

C (7)	prM/M (3)	E (19)	NS1 (11)	NS2A (5)	NS2B (4)	NS3 (9)	NS4A (6)	NS4B (3)	NS5 (43)
I-47-V	prM: V-24-I	N-62-S	I-20-V	M-29-I	I-31-V	I-67-V	M-23-V	S-24-A	I-77-T
I-54-V	M: V-43-A	H-67-N	P-92-S	V-34-M	K-53-R	V-102-A	F-29-L	L-31-F	K-94-R
R-66-K	M: T-47-A	A-83-E	V-95-I	L-47-I	A-76-T	K-105-R	I-52-T	S-120-T	R-106-K
K-69-R		G-191-S	I-176-M	T-146-V	R-125-K	R-258-K	I-57-V		D-107-E
R-81-K		R-207-K	A-217-T	L-215-M		R-396-K	M-66-T		I-131-V
H-102-Q		V-224-M	E-239-D			V-514-I	V-143-I		S-151-P
T-118-V		R-243-K	I-285-V			K-534-R			V-161-M
		D-270-N	I-286-V			S-612-A			A-172-G
		N-271-S	N-289-G			E-613-D			D-176-E
		N-272-K	R-337-K						N-177-S
		V-281-I	E-341-D						V-228-I
		V-318-A							T-229-A
		K-331-R							K-274-R
		I-335-M							E-275-T
		I-344-V							M-289-T
		D-360-E							T-290-A
		S-421-G							S-291-T
		E-450-S							Y-294-H
		A-459-V							K-312-R
									V-321-I
									Y-412-F
									K-440-R
									M-525-L
									D-526-E
									I-553-V
									K-562-R
									Q-566-L
									E-640-D
									S-641-T
									T-644-S
									R-645-K
									T-651-A
									N-565-D
									K-701-R
									N-704-D
									I-727-V
									E-733-D
									I-738-V
									I-800-V
									M-832-T
									V-839-I
									Q-879-K
									K-881-R

**Table 5:** Amino acid sequence of motif 4 within the EDII-EDI hinge region. Virus abbreviations: WNV: West Nile; JEV: Japanese encephalitis; MVEV: Murray Valley encephalitis; SLEV: St. Louis encephalitis; ROCV: Rocio; DENV-1: dengue 1; DENV-2: dengue 2; DENV-3: dengue 3; DENV-4: dengue 4; YFV: yellow fever virus; LGTV: Langat; OHFV: Omsk hemorrhagic fever; TBEV: Tick-borne encephalitis; POWV: Powassan; ZIKV: Zika. For GenBank accession numbers, please see Table 1. The South American YFVs are depicted in blue whereas the African YFVs are red. AA differences among the YFVs are highlighted in yellow.

Virus-Strain	YF Genotype	(260–276)															
YFV—14FA	Angola	A	M	R	V	T	K	D	E	N	D	N	N	L	Y	K	L
YFV—Couma	East and Central Africa	A	M	R	V	T	K	D	E	N	D	N	N	L	Y	K	L
YFV—A-709-A2	East Africa	A	M	R	V	T	K	D	E	N	D	N	N	L	Y	K	L
YFV—Nigeria87B	West Africa I	A	M	R	V	T	K	D	T	N	D	N	N	L	Y	K	L
YFV—FVV	West Africa II	A	M	R	V	T	K	D	T	N	D	N	N	L	Y	K	L
YFV—Asibi	West Africa II	A	M	R	V	T	K	D	T	N	D	N	N	L	Y	K	L
YFV—149/95	South America II	A	M	R	V	T	K	D	T	N	G	S	N	L	Y	K	L
YFV—OBS-7543	South America II	A	M	R	V	T	J	D	T	N	G	S	N	L	Y	K	L
YFV—BeAR51293	South America I	A	M	R	V	T	K	D	T	N	N	S	K	L	Y	K	L
YFV—JSS	South America I	A	M	R	V	T	K	D	T	N	N	S	K	L	Y	K	L
POWV		V	P	L	A	S	V	E	G	Q	-	-	-	K	Y	H	L
LGTV		V	P	V	A	S	I	E	G	T	-	-	-	K	Y	H	L
OHFV		A	P	L	A	H	I	E	G	T	-	-	-	K	Y	H	L
TBEV		V	P	V	A	H	I	E	G	T	-	-	-	K	Y	H	L
ROCV		A	I	P	V	T	V	A	G	T	-	-	-	T	L	T	L
SLEV		A	I	P	A	T	V	S	S	S	-	-	-	T	L	T	L
WNV		A	I	P	V	E	F	S	S	N	-	-	-	T	V	K	L
JEV		A	I	V	V	E	Y	S	S	-	-	-	-	S	V	K	L
MVEV		A	I	P	V	E	F	S	S	S	-	-	-	T	L	K	L
ZIKV		A	L	E	A	E	M	D	G	A	-	-	-	K	G	R	L
DENV-1		A	T	E	I	Q	-	S	G	T	-	-	-	-	T	T	I
DENV-2		A	T	E	I	Q	M	S	S	G	-	-	-	-	N	L	L
DENV-3		A	T	E	I	Q	T	S	G	G	-	-	-	-	T	S	I
DENV-4		A	T	E	V	D	S	G	D	G	-	-	-	-	N	H	M

## Data Availability

Data and materials supporting the findings of this study are available through the NCBI Nucleotide database. All sequences used in this study can be accessed through the GenBank accession numbers in Table 1. NGS data for the strains sequenced in this manuscript can be accessed through: PRJNA1312863.

## Supplementary Materials

The following supporting information can be downloaded at: https://www.mdpi.com/article/10.3390/v18050564/s1, Table S1: Differences between JSS, Brazilian lineage 1B, Asibi, and FVV.
