# Supplementary material for: Genetic Characterization of Yellow Fever Virus Strain JSS, the Original South American Strain
Source: Viruses. 2026 May 15;18(5):564. doi: 10.3390/v18050564 (PMC13211568; doi:10.3390/v18050564)
Supplement: Supplementary file 1 [file viruses-18-00564-s001.zip › viruses-4224253-supplementary.pdf]

Supplementary Table S1: Differences between JSS, Brazilian lineage 1B, Asibi, and FVV

The AA residues which differ between JSS and BeAR512943(Lineage 1B) are shaded light gray.

AA Position in Protein	Asibi (Ghana/1927)	FVV (Senegal/1927)	JSS (Brazil/1935)	BeAR512943 Brazil/1998
C-24	N	N	N	S
C-35	N	N	N	S
C-47	I	I	V	V
C-54	I	I	V	V
C-66	R	R	K	K
C-69	K	K	R	R
C-81	R	R	K	K
C-102	H	H	Q	Q
C-118	T	T	V	V
prM-24	V	V	I	V
M-43	V	V	A	A
M-47	T	T	A	A
E-61	N	N	S	S
E-66	H	H	N	N
E-82	A	A	E	E
E-176	K	K	K	R
E-190	G	G	S	S
E-199	K	T	K	K
E-206	R	R	K	K
E-223	V	V	M	V
E-242	R	R	K	K
E-269	D	D	N	N
E-270	N	N	S	S
E-271	N	N	K	K
E-280	V	V	I	V
E-281	S	S	S	A
E-317	V	V	A	A
E-330	K	K	R	R
E-334	I	I	M	M
E-343	I	I	V	V
E-359	D	D	E	D
E-420	S	S	G	G
E-449	N	N	S	S
E-458	A	A	V	V
NS1-20	I	I	I	V
NS1-92	P	P	S	S
NS1-95	V	V	I	I
NS1-176	I	I	M	M

NS1-217	A	A	T	T
NS1-239	E	E	D	D
NS1-285	I	I	V	V
NS1-286	I	I	V	V
NS1-289	N	N	G	G
NS1-337	R	R	K	R
NS1-341	E	E	D	D
NS2A-29	M	M	I	I
NS2A-34	V	V	M	M
NS2A-47	L	L	I	I
NS2A-118	M	M	M	T
NS2A-146	T	T	V	A
NS2A-215	L	L	M	M
NS2B-31	I	I	V	V
NS2B-53	K	K	R	R
NS2B-76	A	A	T	T
NS2B-125	R	R	K	K
NS3-18	H	H	H	Y
NS3-67	I	I	V	V
NS3-102	V	V	A	A
NS3-105	K	K	R	K
NS3-258	R	R	K	K
NS3-279	V	I	V	V
NS3-396	R	R	K	K
NS3-442	R	R	R	K
NS3-514	V	V	I	I
NS3-534	K	K	R	R
NS3-560	T	T	T	M
NS3-612	S	S	A	A
NS3-613	E	E	D	D
NS4A-4	V	V	V	M
NS4A-23	M	M	V	V
NS4A-29	F	F	L	L
NS4A-52	I	I	T	T
NS4A-57	I	I	V	V
NS4A-66	M	M	T	M
NS4A-118	I	I	I	V
NS4A-126	T	S	T	T
NS4A-143	V	V	I	I
NS4B-19	I	I	I	T
NS4B-24	S	S	A	A
NS4B-31	L	L	F	F
NS4B-120	S	S	T	T
NS5-1	S	R	S	R
NS5-77	I	I	T	T

NS5-94	K	K	R	R
NS5-106	R	R	K	K
NS5-107	D	D	E	E
NS5-131	I	I	V	V
NS5-151	S	S	P	P
NS5-161	V	V	M	M
NS5-172	A	A	G	G
NS5-176	D	D	E	E
NS5-177	N	N	S	S
NS5-228	V	V	I	I
NS5-229	T	T	A	A
NS5-274	K	K	R	R
NS5-275	E	E	T	A
NS5-289	M	M	T	T
NS5-290	T	T	A	A
NS5-291	S	S	T	T
NS5-294	Y	Y	H	H
NS5-312	K	K	R	R
NS5-321	V	V	I	I
NS5-400	I	I	I	M
NS5-412	Y	Y	F	F
NS5-440	K	K	R	R
NS5-525	M	M	L	L
NS5-526	D	D	E	E
NS5-553	I	I	V	I
NS5-562	K	K	R	R
NS5-566	Q	Q	L	L
NS5-640	E	E	D	D
NS5-641	S	S	T	T
NS5-644	T	T	S	T
NS5-645	R	R	K	K
NS5-651	T	T	A	A
NS5-656	N	N	D	D
NS5-701	K	K	R	K
NS5-704	N	N	D	D
NS5-727	I	I	I	V
NS5-733	E	E	D	D
NS5-738	I	I	V	I
NS5-800	I	I	V	V
NS5-832	M	M	T	T
NS5-839	V	V	I	I
NS5-868	L	L	L	F
NS5-879	Q	Q	K	K
NS5-881	K	K	R	R
